# CBX2 is a functional target of miRNA let‐7a and acts as a tumor promoter in osteosarcoma

**DOI:** 10.1002/cam4.2320

**Published:** 2019-05-31

**Authors:** Qicai Han, Chao Li, Yuan Cao, Jie Bao, Kongfei Li, Ruipeng Song, Xiaolong Chen, Juan Li, Xuejian Wu

**Affiliations:** ^1^ Department of Bone and Soft Tissue The First Affiliated Hospital of Zhengzhou University Zhengzhou China; ^2^ Department of Bone and Soft Tissue The Affiliated Cancer Hospital of Zhengzhou University, Henan Cancer Hospital Zhengzhou China; ^3^ Key Laboratory of Clinical Medicine The First Affiliated Hospital of Zhengzhou University Zhengzhou China; ^4^ Department of Hematology Yinzhou People's Hospital affiliated to Medical College of Ningbo University Ningbo China

**Keywords:** CBX2, let‐7a, osteosarcoma, tumor progression

## Abstract

Osteosarcoma is the most common type of primary malignant tumor of skeletal with poor prognosis in children and adolescents. Accumulating evidence indicates that CBX2 is overexpressed in multiple human neoplasm and play a critical role in tumorigenesis and progression. However, its functional role and upstream regulation mechanism in osteosarcoma remain unknown. In the present study, tissue microarray (TMA) analysis was performed to determine the association between CBX2 expression and clinical prognosis of osteosarcoma patients by immunohistochemistry. We also investigated the functional role of CBX2 using small interfering RNA (siRNA) in vitro and in vivo. Additionally, we confirmed the direct binding between CBX2 and let‐7a via qPCR, western blot and luciferase reporter assay. We found that CBX2 is dramatically upregulated in osteosarcoma tissues and high CBX2 expression was correlated with metastasis, recurrence, and chemotherapy response, as well as unfavorable prognosis in patients with osteosarcoma. Similar results were observed in a sarcoma cohort from The Cancer Genome Atlas (TCGA) dataset. Further experiments revealed that CBX2 knockdown significantly impeded osteosarcoma cell proliferation and invasion ability in vitro, and suppressed the tumor growth in tumor xenografts model. Mechanistically, we confirmed that CBX2 is a functional target of miRNA let‐7a. Overexpression of let‐7a inhibits osteosarcoma cell proliferation, which was reversed by CBX2 overexpression. Taken together, our study demonstrates that let‐7a/CBX2 plays a crucial role in osteosarcoma progression. CBX2 could serve as a promising prognostic biomarker and potential therapeutic target for osteosarcoma patients.

## 1 INTRODUCTION

Osteosarcoma is the eighth most common primary malignant skeletal tumor affecting children and adolescent.^1^, ^2^ Despite the emergence of neoadjuvant chemotherapy, the 5‐year survival for osteosarcoma patients with metastasis or recurrence remains poor.^3^ The poor prognosis is due to, at least in part, aggressive biologic behavior and delayed diagnosis.^3^ Therefore, defining the osteosarcoma underlying molecular drivers could facilitate the development of novel strategies target and help guide clinical treatment.

Mounting evidence has shown that there is close relationship between epigenetic alterations and osteosarcoma tumorigenesis.^4^ Recent literature has documented that epigenetic dysregulation induced by the polycomb group (PcG) family protein plays an important role in the development of cancers, including osteosarcoma.^5^ Polycomb repressive complex (PRC) 1 is one of the two main polycomb repressive complexes which assemble from PcG proteins.^6^ The chromobox (CBX2) family, a component of PRC1, is comprised of CBX2/4/6/7/8.^7^ Recent studies have revealed that CBX2 is overexpressed in several cancer type and plays a critical role in tumorigenesis and progression.^8^, ^9^, ^10^ However, the expression pattern and functional role of CBX2 in osteosarcoma and its regulatory mechanisms are still unknown.

MicroRNAs (miRNAs) are a class of extremely conserved non‐protein coding RNAs, which could regulate gene expression at a posttranscriptional level.^11^ Mounting evidence has shown that numerous miRNAs are dysregulated in tumor, contributing to the tumorigenesis and development of almost all cancer, including osteosarcoma.^12^, ^13^, ^14^, ^15^ For instance, miR‐1 and miR‐133b were downregulated in osteosarcoma and may control cell proliferation and cell cycle.^16^ miRNA‐218 was downregulated in osteosarcoma and could suppress cell proliferation and invasion in osteosarcoma.^17^ microRNA let‐7a, a well‐known tumor suppressor in several cancer, was proven to be significantly downregulated in osteosarcoma and could suppress tumor growth via targeting of E2F2.^18^, ^19^ However, the biological roles of let‐7a in osteosarcoma progression remain largely uncertain.

Here we report that CBX2 is markedly upregulated in osteosarcoma and high CBX2 expression predicts poor clinical outcomes in osteosarcoma. Consistent results were observed in sarcoma cohort downloaded from The Cancer Genome Atlas (TCGA). We further revealed the marked positive association between high CBX2 expression and the activation of DNA replication and cell cycle pathway in TCGA sarcoma cohort, indicating that CBX2 may play oncogenic role in osteosarcoma progression through regulating DNA replication and cell cycle. Functional analysis showed that CBX2 silencing osteosarcoma cells exhibited a significantly suppressed proliferation and metastasis capacity. Meanwhile, downregulation of CBX2 suppressed the osteosarcoma tumor xenografts in nude mice. Mechanistically, we confirmed that miRNA let‐7a suppresses CBX2 mRNA expression by directly binding to the 3’untranslated regions of CBX2. Overexpression of let‐7a inhibits osteosarcoma cell proliferation, while increasing CBX2 expression reverses this effect. Taken together, our study demonstrates the importance of let‐7a/CBX2 axis in osteosarcoma progression and CBX2 might be exploited as a potential target for cancer therapy.

## 2 MATERIALS AND METHODS

### 2.1 Patients and specimens

Eighty‐five osteosarcoma specimens and 40 normal specimens from the regions around cancers with completed follow‐up information were collected from The Affiliated Cancer Hospital of Zhengzhou University (Zhengzhou, China) between January 2000 and June 2015. The project was approved by the ethics committee. All patients provided written informed consent under an institutionally approved protocol.

### 2.2 Construction of tissue microarray (TMA) and immunohistochemical (IHC) staining

The osteosarcoma TMA was constructed with all the 65 osteosarcoma specimens and 40 normal specimens as described previously.^20^, ^21^ In brief, for each patient, 0.75‐mm diameter core of the tissue was punched from FFPE tissues and arranged into the TMA blocks. For IHC, paraffin‐embedded tissue sections were processed for IHC using standard procedures. After incubation with primary antibody at 4°C overnight, the slides were then probed with secondary antibody. Then, the sections were dehydrated, cleared with xylene, and mounted with resinene. Two experienced pathologists analyzed the IHC results independently. CBX2 staining was scored from 1+ to 5+ according to staining intensity. Scores of 1+ and 2+ were definitized as CBX2 low expression, while 3+, 4+, and 5 +were definitized as high expression for statistical analysis. The antibody for IHC used in this study is shown in Table S1.

### 2.3 RNA extraction and quantitative realtime PCR

Total RNA was extracted using Trizol reagent (Invitrogen, USA) and then reversely transcribed to cDNA using cDNA reverse transcription kits (Roche, Germany). Then RNA concentration was determined by Nanodrop (Invitrogen, USA). We performed qRT‐PCR using SYBR Green Master (Roche, Germany) and a BIO‐RAD C1000 Thermal Cycler. The relative fold change in expression with respect to a control sample was calculated using the comparative Ct method (2^−△△Ct^) with normalization to GAPDH.

### 2.4 Cell lines and cell culture

Four human osteosarcoma cell lines (MG63, U2OS, Well5 and 143B) and two normal osteoblast cell line (HOBC and HFOB) were stored in our lab. All cells were maintained in DMEM or RPMI 1640 medium supplemented with 10% fetal bovine serum and 100 U/ml penicillin/streptomycin in a humidified incubator at 37°C with 5% CO_2_.

### 2.5 Western blotting assay

Western blot analysis was performed as previously reported.^22^ Briefly, total cell protein lysates were separated by 10% SDS‐PAGE and transferred to a nitrocellulose membrane (Beyotime, China). After incubation with specific antibodies and 5% skim milk powder, the NC membranes were then incubated with specific primary antibodies, followed by HRP‐labeled secondary antibody (Beyotime, China) and detected by chemiluminescence. Quantification analysis of western blot was conducted with ImageJ (Bethesda, USA). The antibodies for western blot used in this study are shown in Table S1.

### 2.6 Cell transfection

siRNA‐targeting CBX2 (CBX2‐siRNA) and negative control (NC) were provided by RiboBio, and the transfection was performed using lipofectamine™ 3000 (Invitrogen, USA) according to the manufacturer's instructions. The lentivirus‐sh‐CBX2 (sh‐CBX2) and negative control (sh‐NC) were all provided by Hanbio (Shanghai, China). Before transfection, cells (1 × 10^5^) were cultured until 80% confluence. The vectors or sh‐CBX2 were transfected, respectively, into osteosarcoma cell lines according to the manufacturer's protocol. Then cells were cultured with 1.5 g/mL puromycin (Invitrogen, USA) for 36 hours for selection.

### 2.7 Cell proliferation and invasion assays

Cell proliferation ability was determined by CCK‐8 assay (Beyotime, China). Briefly, a total of approximately 5000 cells were seeded into 96‐well plates in 100 μL medium per well. The absorbance at 490 nmol/L was assessed after transfection. Cell invasion ability was determined using Transwell with Matrigel (Corning, USA). For colony formation ability assay, approximately 1500 cells per well were seeded in 6‐well plates. After incubating for 15 days, colonies were fixed and stained with 0.5% crystal violet, then the number of colonies was counted.

### 2.8 Luciferase activity analysis

Luciferase activity analysis was performed using the Dual‐Luciferase Reporter Assay System (Promega, USA) as previously reported.^23^, ^24^ Briefly, cells were co‐transfected with 200 ng plasmid containing wild‐type or mutated vectors, as well as 60 nmol/L let‐7a. After 48 hours, luciferase enzyme activity was determined through normalized to firefly luciferase activity.

### 2.9 In vivo tumor growth

Two male BALB/c nude mice (4‐6‐week) were used in the present study. Well5 cells (5×10^6^) infected with shRNA‐CBX2 or negative control shRNA were subcutaneously injected into nude mice. Mice were imaged with an IVIS living imaging system (Caliper, USA) every week. Tumor volume was measured every 7 days and was calculated by the formula: volume (mm3) = (length × width^2^)/2. All animal procedures were approved by Animal Care Committee of The First Affiliated Hospital of Zhengzhou University.

### 2.10 TCGA data analysis

The mRNA gene expression data of sarcoma cohort were downloaded from TCGA LIHC dataset (https://tcga-data.nci.nih.gov/tcga/, The Cancer Genome Atlas). The follow‐up clinical information was available for 257 sarcoma patients, and was utilized to analyze the correlation between CBX2 expression and clinicopathologic features. The raw data were processed and analyzed by BRB‐array tools.

### 2.11 Statistical analysis

Continuous variables are presented as the mean ± SEM from at least three independent experiments. Paired or unpaired student's *t* test (two‐tailed) was used to compare the differences between two groups, respectively. A chi‐squared test was used to evaluate the association between CBX2 expression and clinicopathological parameters. The survival rates were determined using the Kaplan‐Meier method (log‐rank test). *P* value of <0.05 was considered significant. Statistical analysis was performed using SPSS version 23.0 (SPSS Inc, USA).

## 3 RESULTS

### 3.1 CBX2 is overexpressed and correlated with poor prognosis in osteosarcoma

**Figure 1:**
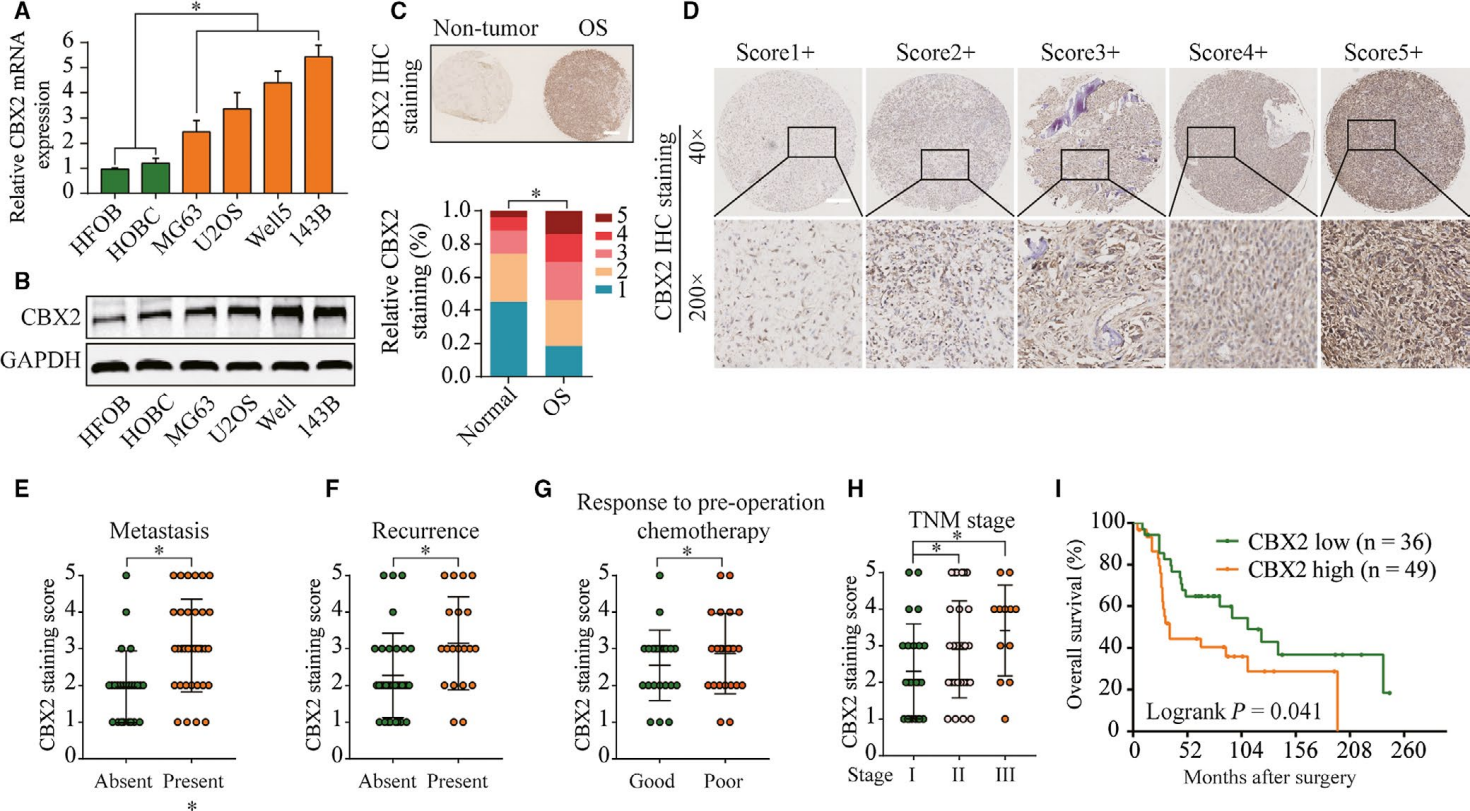
Overexpression of CBX2 was correlated with prognosis of osteosarcoma patients. The expressions of CBX2 mRNA (A) and protein (B) in osteosarcoma cell lines (MG63, U2OS, Well5, and 143B) and normal human osteoblast cell lines (HOBC and HFOB), the scale bar is 200 µm. (C) Distribution of CBX2 immunohistochemical staining scores in osteosarcoma tissues and normal tissues. D, Representative CBX2 immunohistochemical staining patterns with different staining scores in osteosarcoma tissues. B, Distribution of CBX2 IHC staining scores in osteosarcoma tissues according to metastasis, recurrence status, chemotherapy response, and tumor‐node‐metastasis stage (TNM) classification. C, Kaplan‐Meier overall survival analysis between expression of CBX2 (yellow, high CBX2 expression; green, low CBX2 expression)

Recent literatures have revealed that CBX2 is overexpressed in multiple human neoplasm. To determine the expression and potential functions of CBX2 in osteosarcoma, we first analyzed the CBX2 expression in normal osteoblast cells (HFOB and HOBC) and several osteosarcoma cell lines. We found that CBX2 was a significant higher expression in osteosarcoma cells than normal osteoblast cells (Figure 1A,B). Consistently, the rate of CBX2 staining was significantly increased in osteosarcoma tissues compared with normal tissues, while CBX2 immunoreactivity was observed primarily in the cell cytoplasm (Figure 1C,D). We then determined the clinical relevance of CBX2 in osteosarcoma, we found that CBX2 expression was progressively increased gradually during osteosarcoma progression in patients with metastasis or recurrence (Figure 1E,F). In addition, high CBX2 staining was significantly increased in patients with poor response to preoperation to chemotherapy or advanced clinical stage (Figure 1G, H; Table 1). Moreover, Kaplan‐Meier analysis revealed that upregulated CBX2 expression was correlated with worse overall survival in patients with osteosarcoma (Figure 1I). Furthermore, multivariate Cox regression analysis showed that upregulated CBX2 was an independent prognostic factor for poor prognosis (Table2). Altogether, these findings indicated a close association between CBX2 overexpression and worse osteosarcoma prognosis, and suggest that CBX2 may function as an oncogene in the development of osteosarcoma.

**Table 1:** Correlation of clinicopathological features with CBX2 expression in osteosarcoma cohort

Clinicopathological features	Variables	CBX2 expression		*P value*
		Low expression (n = 39)	High expression (n = 46)	
Age (years)	≤65	20 (51.3)	26 (56.5)	0.629
	>65	19 (48.7)	20 (43.5)	
Gender	Male	25 (64.1)	28 (60.9)	0.759
	Female	14 (35.9)	18 (39.1)	
Tumor site	Femur	17 (43.6)	22 (47.8)	0.875
	Tibia	13 (33.3)	13 (28.3)	
	Other	9(23.1)	11 (23.9)	
TNM stage	Stage I	21(53.8)	12 (26.1)	**0.028**
	Stage II	10(25.6)	16 (34.8)	
	Stage III	8(20.5)	18 (39.1)	
Metastasis	Absent	22(56.4)	16 (34.8)	**0.047**
	Present	17(43.6)	30 (65.2)	
Recurrence	Absent	25(64.1)	19 (41.3)	**0.036**
	Present	14(35.9)	27 (58.7)	
Chemotherapy response	Good	19	12	**0.038**
	Poor	10	20	
	NA	11	14	
Tumor size	≤7.5 cm	26 (66.7)	24 (52.2)	0.176
	>7.5 cm	13 (33.3)	22 (47.8)	
Survival	Live	29 (74.4)	21 (45.7)	**0.007**
	Dead	10 (25.6)	25 (54.3)	

Bold values indicate statistical significance, *P* < 0.05.

**Table 2:** Correlation of clinicopathological features with CBX2 expression in osteosarcoma TMA cohort

	Univariate analysis			Multivariate analysis		
	HR	95% CI	*P* value	HR	95% CI	*P* value
Univariate and multivariate analysis of overall survival in osteosarcoma patients (n = 85)						
Age	1.115	0.660‐1.988	0.456			
Gender	0.717	0.433‐1.390	0.528			
Tumor site	1.122	0.608‐1.991	0.347			
TNM stage	2.033	1.244‐3.175	0.024	1.509	0.842‐2.008	0.058
Metastasis	2.457	1.662‐3.621	0.011	2.300	1.333‐3.174	0.015
Tumor size (cm)	1.132	0.629‐2.154	0.063			
Recurrence	2.032	1.252‐3.099	0.017	1.618	0.906‐2.513	0.031
Chemotherapy response	1.452	0.801‐1.791	0.067			
CBX2 expression	2.236	1.368‐3.357	0.012	1.914	1.200‐3.019	0.014
Univariate and multivariate analysis of disease‐free survival in osteosarcoma patients (n = 85)						
Age	1.001	0.622‐1.909	0.733			
Gender	0.866	0.581‐1.619	0.819			
Tumor site	1.189	0.786‐2.276	0.346			
TNM stage	2.158	1.313‐3.228	0.075			
Metastasis	3.004	1.917‐5.519	0.002	2.448	1.446‐3.813	0.037
Tumor size (cm)	2.019	1.254‐3.680	0.080			
Recurrence	2.032	1.263‐3.617	0.077	2.622	1.706‐3.912	0.031
Chemotherapy response	1.357	0.783‐1.649	0.852			
CBX2 expression	5.225	2.547‐10.566	<0.001	2.915	1.804‐5.319	0.002

### 3.2 High CBX expression was positively associated with malignancies in sarcoma

**Figure 2:**
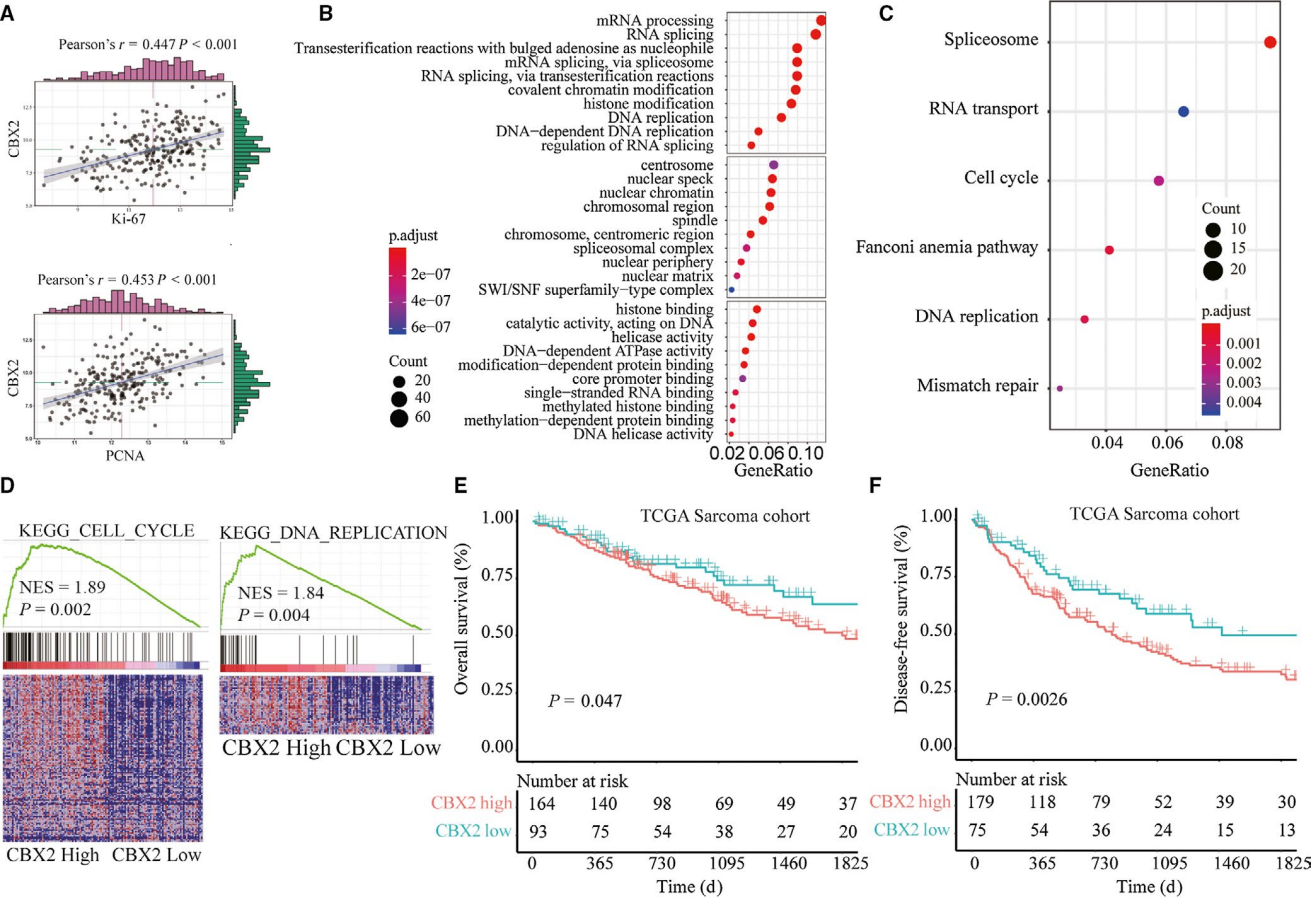
Functional and pathway enrichment analysis of CBX2 in TCGA sarcoma cohort. A, Correlation analysis between the expression of CBX2, Ki‐67, and PCNA. B, gene ontology (GO) enrichment and (C) KEGG enrichment analysis of the top 800 genes with highest CBX2 correlation coefficient. D, The Gene Set Enrichment Analysis from the TCGA sarcoma dataset revealed a high expression of CBX2 correlated with gene signatures of DNA replication and cell cycle. E, Kaplan‐Meier overall survival analysis between different degrees of CBX2 expression. F, Kaplan‐Meier relapse‐free survival analysis between different degrees of CBX2 expression.

To explore the underlying mechanisms of CBX2 involved in osteosarcoma progression, we conducted bioinformatics analysis based on the sarcoma gene expression dataset from TCGA database. CBX2 expression level was positively associated with the expression of PCNA and Ki67, two proliferation markers of neoplasm (Figure 2A). Additionally, we performed gene ontology (GO) enrichment analysis and pathway enrichment analysis of the top 800 genes with highest CBX2 correlation coefficient. The results showed that cell cycle and DNA replication pathways were significant related to the CBX2 overexpression. Consistently, gene set enrichment analysis (GSEA) analysis showed that cell cycle and DNA replication were two overwhelmingly enriched genesets highly related with CBX2 overexpression (Figure 2C,D), indicating that CBX2 might contribute to progression of sarcoma through regulating cell cycle and DNA replication.

Furthermore, all sarcoma patients were divided into high CBX2 expression group and low CBX2 expression group with the best cutoff chosen by xtitle software. Intriguingly, high CBX2 expression was discovered to correlate with poorer overall survival (OS) rate (*P* = 0.047), (Figure 2E) and shorter progression‐free survival (PFS) period (*P* = 0.0026; Figure 2F). These findings suggested that CBX2 overexpression may be a common feature of sarcoma and could promote the clinical progression of sarcoma.

### 3.3 CBX2 promotes osteosarcoma cell proliferation and metastasis in vitro

**Figure 3:**
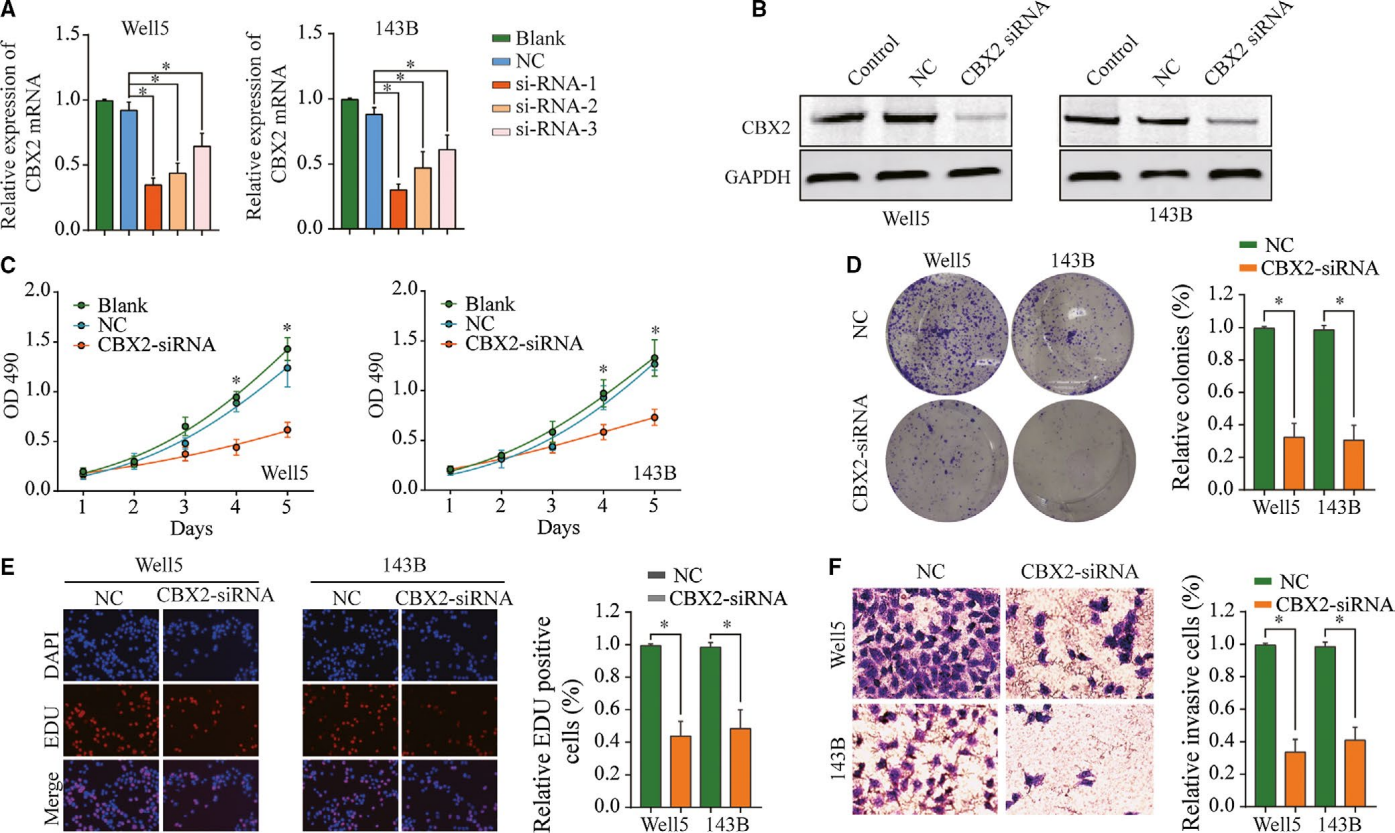
Inhibiting CBX2 suppresses osteosarcoma cell line proliferation and invasion. A, CBX2 mRNA expression was decreased after transfection with siRNA‐targeting CBX2. B, CBX2 protein expression was decreased after transfection with siRNA‐targeting CBX2. C, CBX2 silencing inhibited proliferation of well5 and 143B cells. D and E, CBX2 knockdown inhibited cell proliferation in osteosarcoma cells, as determined by colony formation and EDU assays, respectively. F, The invasiveness of well5 and 143B cells infected with CBX2 siRNA was significantly suppressed according to cell invasion assay. *P < 0.05

To explore the biological function of CBX2 in osteosarcoma cells, qtPCR and western blot analysis confirmed low mRNA expression of CBX2 in well5 and 143B cell lines after CBX2‐siRNA transfection (Figure 3A,B). We then chose the siRNA with highest knockdown effective for further experiments. Next, cell counting kit‐8 (CCK‐8) assays were conducted to assess the influence of CBX2 silencing on osteosarcoma cell proliferation. Compared with NC, well5 and 143B cells transfected with CBX2 siRNA had a significant decrease in cell viability (Figure 3C). Similar results were observed in colony formation assay (Figure 3D). Furthermore, we found that CBX2 silencing could significantly decrease EdU incorporation rate in osteosarcoma cells via EdU incorporation assays (Figure 3E). Moreover, we examined whether CBX2 knockdown could influence the invasion ability of osteosarcoma cells by Matrigel invasion assay. As shown in Figure 3F, invasive cells were remarkably decreased after CBX2 knockdown. In addition, we also analyzed the function of CBX2 in proliferation of normal human osteoblast, and the results of CCK‐8, colony formation, and EDU assay showed that suppression of CBX2 could not affect proliferation of normal human osteoblast cell lines (Figure S1).These data demonstrated that CBX2 could promote the proliferation and invasion of osteosarcoma cells.

### 3.4 Silencing CBX2 represses osteosarcoma tumorigenesis in vivo

**Figure 4:**
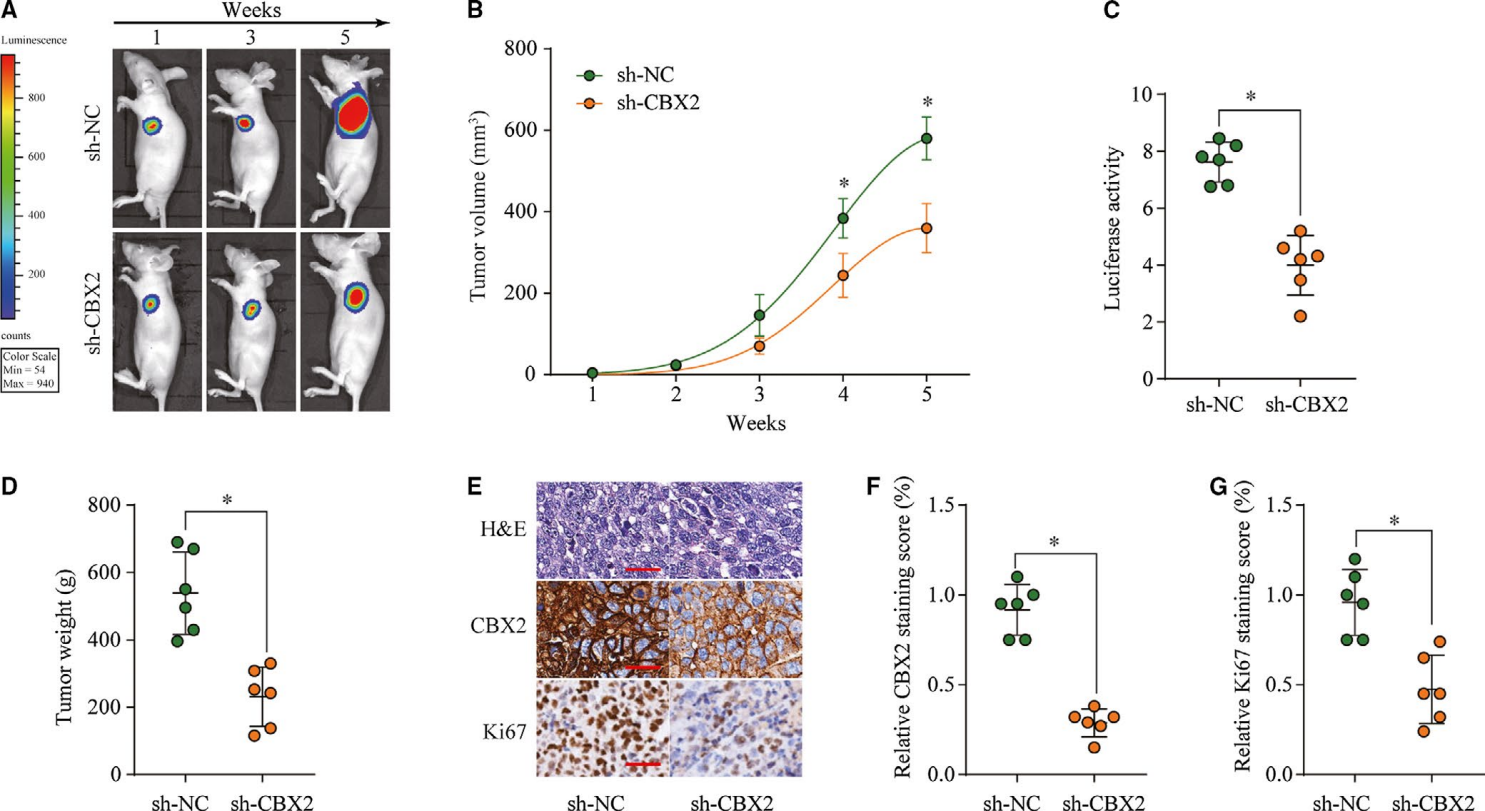
CBX2 promotes osteosarcoma progression in vivo. A, Detecting the luciferase signal of tumor by living imaging system. B, Tumor volume in sh‐CBX2 group was significantly lower than those in sh‐NC group. C, The luciferase activity in the sh‐CBX2 group was lower than in the sh‐NC group. D, Tumor weight in sh‐CBX2 group was significantly decreased than those in sh‐NC group. E‐G, CBX2 and Ki‐67 staining of tumor tissues from mice inoculated with sh‐CBX2 and sh‐NC infected well5 cells. The scale bar is 20 µm

To further confirm the in vitro results, we established subcutaneous tumor in BALB/c nude mice using well5 cells transected with negative control shRNA (sh‐NC) or transected with shRNA‐targeting CBX2 (sh‐CBX2). Tumor volume and ex vivo imaging luciferase signal were measured every 7 days. The subcutaneous tumors in the CBX2 silencing group grew dramatically slower than those in the control group (Figure 4A,B). Consistently, the average tumor luciferase activity and tumor weight were significantly decreased after CBX2 knockdown (Figure 4C,D). In addition, CBX2 and ki‐67 were assessed using IHC analysis in xenograft tumor tissues and results revealed that both CBX2 and Ki‐67 staining intensities were markedly decreased in the tumors from the sh‐CBX2 group (Figure 4E–G). The above results strongly suggested that CBX2 might be crucial for the osteosarcoma cell tumorigenicity.

### 3.5 Prediction of CBX2 as a target gene of miRNA let‐7a

**Figure 5:**
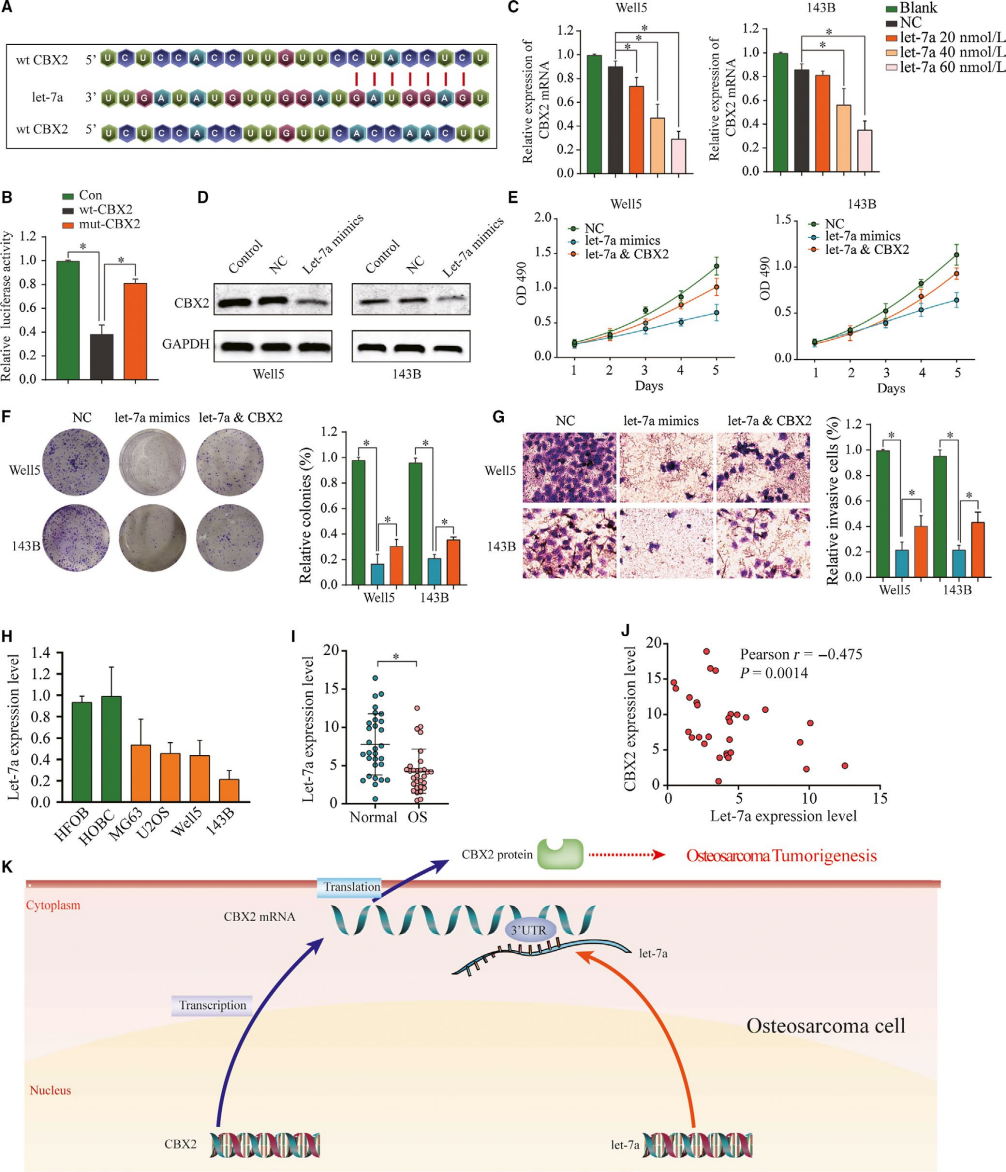
CBX2 as the functional target of miRNA let‐7a. A, Schematic representation of the let‐7a binding site in CBX2 3’‐UTR. B, Representative luciferase activity in cells co‐transfected with wild‐type or mutated reporter plasmids after ectopic expression of let‐7a. The effect of let‐7a mimics or NC on the expression of CBX2 in well5 and 143B cells was determined by (C) qtPCR and (D) Western blot. E, Overexpression of let‐7a inhibited the proliferation ability of well5 and 143B cells, which was partially reversed by CBX2 overexpression. F and G, Colony formation and invasion assay indicated that compared to negative control groups, let‐7a overexpression significantly inhibited proliferative and invasion activity, respectively, which was significantly reversed by the CBX2 overexpression. *P < 0.05. H and I, The expression of let‐7a was detected in both tissue (osteosarcoma tissue and adjacent normal tissue) and cellular levels (HFOB, HOBC, MG63, U2OS, Well5, and 143B cells). *P < 0.05. J, relationship between let‐7a expression and CBX2 expression in human osteosarcoma tissues was analyzed (r=−0.475, *P* = 0.0014)

Previous studies have revealed that numerous miRNAs are dysregulated and contributed to the initiation and development of the osteosarcoma; we hypothesized that miRNA might be involved in the mechanism of CBX2 dysregulation in osteosarcoma. To explore the molecular mechanism underlying CBX2 deregulation via miRNA regulation, computational algorithms (TargetScan and miRanda) were used in combination to search for miRNAs which could bind to the 3’‐UTR of CBX2. According to the comprehensive analysis results, miRNA let‐7a, a well‐known tumor suppressor, was chosen for further validation. The predicted interaction between let‐7a and the target site in the CBX2 3’‐UTR is illustrated in Figure 5A. Subsequently, luciferase reporter analysis was conducted to confirm the directly targeting between let‐7a and CBX2. Luciferase activity was dramatically decreased in cells overexpressing let‐7a when transfected with the wild reporter plasmid CBX2 3’‐UTR, but not in mutant‐type CBX2 or NC group, indicating the specificity binding between let‐7a and CBX2 (Figure 5B). Additionally, we transfected let‐7a mimics into well5 and 143B cells and qPCR results showed that CBX2 was downregulated in well5 and 143B cells treated with let‐7a mimics (Figure 5C). Consistent results were observed in osteosarcoma cell lines by western blot (Figure 5D). This finding demonstrated that let‐7a directly binds to the 3’‐UTR of the CBX2 transcript to suppress CBX2 expression.

### 3.6 Let‐7a's tumor suppression activity is in part through targeting CBX2

Several studies reported that let‐7a could inhibit osteosarcoma cell growth. To confirm whether let‐7a suppresses the osteosarcoma cells proliferation through targeting CBX2, we performed loss‐ and gain‐of‐function experiment in osteosarcoma cell lines. Initially, we introduced CBX2 gene or let‐7a mimics into well5 and 143B cells. Proliferation assays indicated that let‐7a overexpression significantly inhibited the growth ability of well5 and 143B cells, which was partially reversed by overexpression of CBX2 (Figure 5E). Consistently, result of colony formation experiment certified that unregulated let‐7a suppressed the proliferative activity of osteosarcoma cells, which was significantly reversed by overexpression of CBX2 (Figure 5F). Additional, restoration of CBX2 expression significantly reversed the inhibitory effects of let‐7a on cell invasion (Figure 5G).Furthermore, we compared the expression of let‐7a between human osteosarcoma and normal osteoblast at both tissue and cellular levels, and observed that let‐7a level was lower in osteosarcoma tissues and cell lines than normal osteoblast (Figure 5H–I). Linear correlation analysis showed that the expression of CBX2 was negative correlated with let‐7a expression in osteosarcoma tissues (Figure 5J). The above findings support the idea that CBX2 plays a crucial role in the mechanisms underlying the tumor‐suppressive functions of let‐7a in osteosarcoma.

## 4 DISCUSSION

CBX2 could recruit PRC1 proteins to mitotic chromosomes and then exhibit the function of histone modification and transcriptional regulation.^7^ In addition, PRC1 promotes cancer cell proliferation through regulating the PcG activity in cancer. Recent study has revealed that CBX2 was significantly upregulated in breast cancer and prostate cancer and may serve as a prognostic biomarker.^8^, ^9^, ^10^ Consistent with this previous study, our study confirmed the dramatically upregulated CBX2 in osteosarcoma and high CBX2 expression was correlated with metastasis, recurrence, and chemotherapy response, as well as unfavorable prognosis in patients with osteosarcoma. Given that osteosarcoma and soft tissue sarcomas might share a similar pathogenesis and etiology,^25^ as well as the lack of large cohort of osteosarcoma expression profile, we analyzed the correlation of CBX2 with sarcoma patient survival to further confirm our finding in osteosarcoma with relative large cohort in TCGA dataset. Consistent with the survival analysis results in osteosarcoma, upregulated CBX2 expression was correlated with unfavorable prognosis in patients with sarcoma. These results demonstrated that CBX2 may function as an oncogene in the progression of osteosarcoma, and CBX2 could be served as a potential prognostic biomarker.

To detect the biological function of CBX2 on osteosarcoma progression, we disrupted CBX2 expression in osteosarcoma cells and the results showed that the CBX2 silencing markedly inhibited the tumor proliferation in vitro and tumorigenesis in vivo. Additionally, we have also shown that CBX2 expression is dramatically elevated in metastatic tumors compared to metastatic absent patient. Consistent with this notion, invasive ability of osteosarcoma cell was dramatically suppressed following CBX2 knockdown. In agreement with our results, Clermont et al reported that CBX2 silencing could inhibit cell proliferation and metastasis in prostate cancer. Moreover, CBX2 could regulate the expression of key genes involved in cancer proliferation and metastasis.^9^ These results are consistent with weaken proliferative features of CBX2‐deficient animals.^26^ Additionally, CBX2 silencing could cause significant downregulation of several proteins involved in mitotic spindle assembly, indicating the close correlation between CBX2 expression and cell cycle.^26^ Meantime, CBX2 could directly affect cell cycle progression through its regulation with condensed chromatin.^7^ Through bioinformatics analysis, we revealed that high expression of CBX2 was associated with gene signatures of cell cycle and DNA replication, in line with its reported role in cellular proliferation. Taken together, these finding indicated that CBX2 might function as an oncogene in human osteosarcoma likely through regulating cell cycle and DNA replication, and CBX2 may serve as a putative therapeutic target in osteosarcoma.

After validating CBX2 that acts as an oncogene, we confirmed that miRNA let‐7a could directly target CBX2 in osteosarcoma cell lines through bioinformatics prediction and experimental validation. let‐7a is proven to be a significantly decreased miRNA in different tumors. As a tumor suppressor miRNA, let‐7a was found to be downregulated in osteosarcoma tissues than in the normal bone tissues,^18^ and let‐7a could suppress the cancer cell proliferation through inhibiting it's downstream oncogenes such as RAS, CCR7, E2F2, and CCDN2.^7^, ^27^, ^28^ Here, we show that let‐7a targets CBX2 as well and represses its expression. More importantly, the inhibition effects of let‐7a on proliferation in osteosarcoma were reversed by the CBX2 overexpression. Taking the functions of CBX2 and let‐7a contributed by our and others' endeavors into account, we reveal a novel mechanism for let‐7a as a tumor suppressor via targeting CBX2 in the progression of osteosarcoma (Figure5K).

## 5 CONCLUSION

In conclusion, we determined that CBX2 is upregulated in osteosarcoma and might serve as a prognostic factor for osteosarcoma. Knockdown of CBX2 decreased the proliferation and invasion of osteosarcoma cells. Moreover, CBX2 is a direct functional target of let‐7a. The CBX2/let‐7a axis provides novel insight into the mechanisms underlying osteosarcoma progression and may serve as a novel therapeutic target for osteosarcoma.

## CONSENT FOR PUBLICATION

The participant gave informed consent before taking part in this study. All samples were de‐identified.

## ETHICS APPROVAL AND CONSENT TO PARTICIPATE

The study was approved by the human ethics committee of the First Affiliated Hospital of Zhengzhou University. All patients signed the written informed consent. All the animal experiments were approved by the ethics committee of the First Affiliated Hospital of Zhengzhou University.

## CONFLICT OF INTEREST

The authors confirm that there are no conflict of interest.

## AUTHORS CONTRIBUTIONS

QCH and CL performed the cell in vitro experimental work. XC and JL participated in data analysis. XLC and ZGR designed and performed the animal experiment. YGL and GYC conceived the study and participated in its design. The manuscript was written by XJW and QCH. All authors checked and approved the final manuscript.

## DATA AVAILABILITY STATEMENT

Available under request.
