# Supplementary figures and images for: CBX2 is a functional target of miRNA let‐7a and acts as a tumor promoter in osteosarcoma
Source: Cancer Med. 2019 May 31;8(8):3981–91. doi: 10.1002/cam4.2320 (PMC6639449; doi:10.1002/cam4.2320)

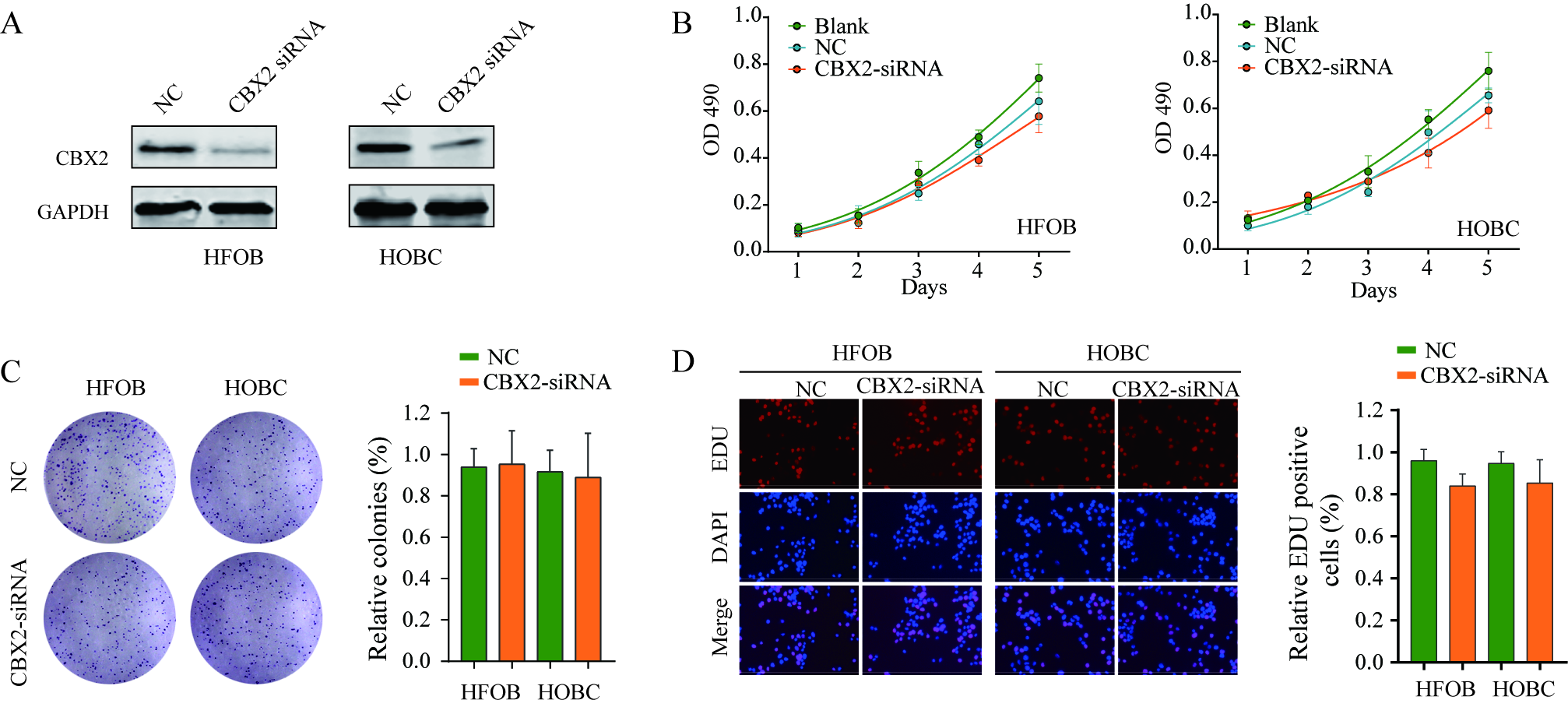

Supplement: Supplementary file 1 [file CAM4-8-3981-s001.tif]
