# Supplementary material for: CBX2 is a functional target of miRNA let‐7a and acts as a tumor promoter in osteosarcoma
Source: Cancer Med. 2019 May 31;8(8):3981–91. doi: 10.1002/cam4.2320 (PMC6639449; doi:10.1002/cam4.2320)
Supplement: Supplementary file 2 [file CAM4-8-3981-s002.docx]

**Supplementary Table 1. Information on antibodies used in this study**

| **Antibody** | **WB** | **IHC** | **Specificity** | **Company** |
| --- | --- | --- | --- | --- |
| GAPDH | 1:5000 | / | Mouse monoclonal | Proteintech Group, China |
| CBX2 | 1:500 | 1:100 | Rabbit monoclonal | Proteintech Group, China |
| Ki-67 | / | 1:200 | Rabbit monoclonal | Proteintech Group, China |
